# Evolutionary development and morphological modifications of the brain: an interview with Angelika Stollewerk

**DOI:** 10.1186/s12915-018-0590-8

**Published:** 2018-11-01

**Authors:** Angelika Stollewerk

**Affiliations:** School of Biological and Chemical Sciences, Queen Mary University of London, Mile End Road, London, E1 4NS UK

**Keywords:** Evo-devo, Evolution, Nervous system, Arthropod, Evolution of sociality, Cell atlases, Neurogenesis

## Abstract

Angelika Stollewerk is a Reader at Queen Mary University of London, where her lab uses a diverse range of species to study the evolution of the arthropod nervous system. Angelika spoke to us about social spiders, the future of evo-devo, and open peer review.

## What are your current research interests?

I have lots of different interests but they all revolve around arthropods and the evolution of the nervous system. I like to work with many different species and one exciting new project involves two social spider species, which we have recently collected. We are looking for links between peripheral nervous system evolution and the evolution of sociality using gene expression and functional and behavioural approaches.

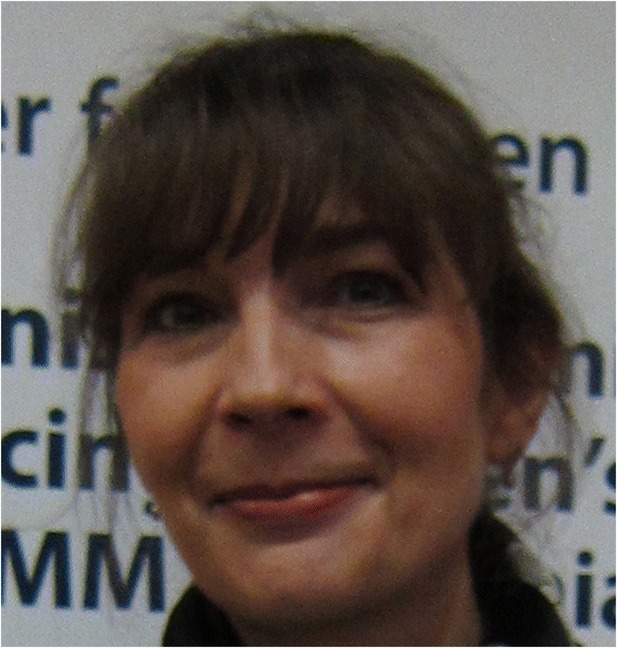



## What are your predictions for the field over the next 5 years?

The evo-devo research field has changed substantially over the past 10 years. Rather than analyzing single genes, morphology and a handful of representatives for taxa/phyla, the field has moved on to generating transcriptomes of developmental stages, tissues and single cells and substantially increased the genome coverage, in particular in arthropods (e.g., [1–7]). One of the next steps (in my area) will be to systematically establish cell atlases for a large number of species across all phyla to identify evolutionary cell lineages and work out the different levels of evolutionary relationships between tissues, organs and cells.

## What motivates you to provide peer review for journals?

I like reading brand new research! I also think peer review is an essential service to the academic community to ensure high quality publications and this responsibility should be shared by as many researchers as possible.

## What changes, if any, would you make to the current system of peer review?

All journals should change to open peer review.

### Website:

http://astollewerk.sbcs.qmul.ac.uk/index.html and http://www.sbcs.qmul.ac.uk/staff/angelikastollewerk.html.
